# Calcifediol Cornerstone of the Vitamin D Endocrine System

**DOI:** 10.3390/nu15102290

**Published:** 2023-05-12

**Authors:** Jose Manuel Quesada-Gomez, Roger Bouillon

**Affiliations:** 1 Instituto Maimónides de Investigación Biomédica de Córdoba (IMIBIC), Hospital Universitario Reina Sofía, 14004 Córdoba, Spain; jmquesada@uco.es; 2 Centro de Investigación Biomédica en Red de Fragilidad y Envejecimiento Saludable (CIBERFES), Instituto de Salud Carlos III, 28029 Madrid, Spain; 3 Clinical and Experimental Endocrinology, Department of Chronic Diseases and Metabolism, Catholic University of Leuven, 3000 Leuven, Belgium

It is likely that rickets has afflicted humanity since the dawn of time, but it was first described in great detail in the mid-17th century. Its etiology was not unraveled until little over a century ago with the discovery of vitamin D. Since then, the supplementation of “vitamin” D in the form of cod liver oil, cholecalciferol (“vitamin” D3) or ergocalciferol (“vitamin” D2), in order to cure or prevent endemic nutritional rickets, has been widely implemented and proven to be very effective. However, cholecalciferol is not really a “vitamin” D because it can be synthesized in the skin by UV-B rays. It is a threshold nutrient of the “vitamin” D endocrine system (VDES), and is comparable to iodide in the thyroid hormone system [1].

Cholecalciferol/”vitamin” D3, a nutrient of the VDES, is inactive and requires two sequential hydroxylations, one at carbon 25 and the other at 1α, to generate first 25-hydroxyvitamin D3 (25OHD/calcifediol), which is similar to thyroxine/T4, and then to form 1α, 25 (OH)2D/calcitriol, the active hormone of the system, which is similar to Triiodothyronine/T3 [1]. These are absolutely essential hydroxylations, as evidenced by the mutations that encode these genes in humans and animals, thus causing rickets types A1 and A2 [1,2,3].

Calcifediol (25 OHD) binds with high affinity to a specific serum transporter protein, DBP, and thus facilitates the availability of large quantities of circulating precursors with a long half-life, similar to thyroxine (2 and 1 week, respectively). The transient loss of nutrient supply (vitamin D/iodine) does not immediately imply that the actual ligand does not have a hormonal effect regarding its nuclear receptor [1, 25 (OH) 2D] and VDR. Calcifediol is a substrate that synthesizes 1α, 25-dihydroxyvitamin D3 [1α, 25 (OH) 2D] or calcitriol, which is mediated by the CYP27B1 gene. This metabolite is a hormonally active form of the system (similar to T3 in thyroid hormones) that has a high affinity for its nuclear receptor (VDR) and activates or represses a large number of genes in all vertebrates, including humans. Calcifediol is inactivated into other metabolites such as 24R, 25 (OH) 2D3 (similar to reverse T3), which is mediated by the CYP24A1 gene. The VDR and enzymes employed in the activation and catabolism of “vitamin” D are expressed in most cells and tissues of the body, with multiple extra-skeletal actions occurring in addition to the well-known and classical skeletal ones [1].

It is therefore not surprising that the measurement of calcifediol in serum is used to assess the nutritional status of the system [1,2,3], although this measurement is often expressed and erroneously described as “vitamin” D levels. In this regard, and to cause further nosological confusion, vitamin D itself, as well as calcifediol or even calcitriol, are often referred to as vitamin D [4].

In recent decades, the volume of publications on various aspects of VDES has expanded exponentially, confirming its role in calcium homeostasis and the maintenance of skeletal integrity. Numerous epidemiological studies have associated a deficient calcifediol/25 (OH) D status with a risk of cancer and chronic diseases, including autoimmune, cardiovascular, neurological or infectious diseases, and ultimately with an increased risk of mortality [1]. This information has led to a widespread belief among specialists, the media, patients, and the general population that the indiscriminate supplementation of the majority of the population with “vitamin” D (cholecalciferol or ergocalciferol) is an appropriate and desirable therapeutic option due to its health benefits, thus generating multiple hypotheses and more than 3000 randomized clinical trials (RCTs) registered in the NIH TrialNet [5].

However, several recent large RCTs evaluating crucial bone and extra-skeletal endpoints have suggested that the effects of “vitamin” D are trivial or non-existent, generating a great deal of controversy. Vitamin D administration in the VIDA, VITAL, D2d or D-Health trials does not appear to have had a protective effect against falls or fractures [6], cardiovascular disease [7,8], cancer [8], the progression of pre-diabetes to diabetes (at least in the intention-to-treat analysis) [9], or in improving the prognosis of patients admitted to intensive care units [10], although the VITAL trial revealed a significant reduction in new autoimmune diseases [11]. Even a large meta-analysis reviewing 81 RCTs and 53,537 patients concluded that “vitamin” D supplementation has no significant effect on fractures, falls or bone mineral density [12]. Considering these findings and their impact on the media, physicians and patients may mistakenly conclude that administering “vitamin” D supplements can be halted as they have no beneficial health effects. This is a potentially dangerous message, given the high prevalence of calcifediol/25 (OH) D deficiency worldwide [3].

The meta-analysis [12] that disqualified bone and the extra-osseous actions of “vitamin” D also included many trials that considered vitamin D-replete patients at baseline, used very high bolus doses of “vitamin” D, or were designed to be too short in duration for their intended purpose. Based on these recent trials and meta-analyses, it is clear that vitamin D supplementation has no clear health benefits if prescribed to already vitamin D-replete subjects. High intermittent bolus doses may be harmful, but monthly doses of 100,000 IU of vitamin D are safe. These trials were not sufficient to study the effects of supplementing vitamin D-deficient subjects with vitamin D, nor the effects of supplementing subjects with a rather poor vitamin D status and/or poor calcium intake at baseline with combined vitamin D and calcium [13].

Calcifediol may well be an alternative to the use of vitamin D supplementation [14,15]. Calcifediol is absorbed by intestinal cells, transported through the portal vein and is, therefore, immediately accessible to the circulation; meanwhile, native vitamin D is transported more slowly by chylomicrons via the lymphatic system. This rapid absorption and its independence from hepatic 25-hydroxylation lead to pharmacokinetics that make calcifediol an immediately available substrate for the synthesis of calcitriol, either for systemic transport, local para or autocrine actions [16,17]. In contrast to native D “vitamins”, calcifediol has a linear and predictable dose–response curve that is independent of baseline serum 25 (OH) D concentrations and is about three times more potent than vitamin D in patients with mild 25 (OH) D deficiency. This potency is 6–8 times higher than vitamin D when the baseline serum 25OHD is higher or when large doses are compared [17]. Thus, clinical trials comparing the efficacy and safety of calcifediol and cholecalciferol in vitamin-deficient situations in the short and long term have reported that calcifediol is more effective; indeed, it sees a more rapid onset of action compared to the monthly administration of cholecalciferol, and possesses no associated safety concerns [14,15]. Long-term calcifediol treatment produces stable and sustained 25 (OH) D concentrations; however, as with “vitamin” D, the withdrawal of treatment leads to a steep decline in 25 (OH) D levels compared to pre-treatment levels [15,18].

This Special Issue “Vitamin D Endocrine System: Calcifediol for Treatment and Prevention of Infection and Disease” defines some guidelines for understanding calcifediol as a cornerstone of the vitamin D system and its (potentially) great utility in case of deficiency. It also addresses a number of other interesting and highly topical questions regarding calcifediol.

J. White reviews the significance of calcifediol in maintaining an adequate innate immune response [19]; ML. Brandi’s group evaluates the rapid non-transcriptional effects of calcifediol [20]; M. Barbagallo et al., in a systematic review and meta-analysis, report that calcifediol may have a positive effect on muscle strength parameters and, with less evidence, on physical performance; they thus propose its potential utility in sarcopenia [21]. JL. Castrillo et al. collect the available evidence and reveal that calcifediol therapy is an excellent option for the treatment of “vitamin” D deficiency, particularly regarding its efficacy and safety [22]. In fact, it has been revealed that the administration of calcifediol may even be the preferred strategy in the case of malabsorptive diseases, after bariatric surgery or in the case of other conditions in which altered 25-hydroxylase activity in the liver is suspected, such as in inflammatory diseases.

Therefore, adequate calcifediol levels ought to be maintained throughout life, as shown by R. Bouillon et al. [3], for female health and fertility, as described by A. Arnanz et al. [23], and in pediatrics, as propounded by L. Castano’s group [2].

Unfortunately, despite being the cornerstone of the vitamin D endocrine system, calcifediol has “paradoxically” only been employed to prevent or treat deficiencies in a few countries, predominantly in Spain, Belgium, or Italy, and only very recently in several other European countries and in Latin America; it has never been utilized in the United States, Canada or the United Kingdom [17]. One exciting new chapter in the understanding of calcifediol has opened with the report of very high levels of calcifediol sulphate being found via tandem mass spectrometry in patients whose calcifediol/25OHD levels, when measured by conventional ELISA, were low [24]. This leads us to ponder several further questions: what is the cause of these levels and, indeed, what is the role of calcifediol sulphate? Are we now dealing with a metabolite in search of a function?
